# Social and Environmental Factors Related to Boys’ and Girls’ Park-Based Physical Activity

**DOI:** 10.5888/pcd12.140532

**Published:** 2015-06-18

**Authors:** Jason N. Bocarro, Myron F. Floyd, William R. Smith, Michael B. Edwards, Courtney L. Schultz, Perver Baran, Robin A. Moore, Nilda Cosco, Luis J. Suau

**Affiliations:** Author Affiliations: Myron F. Floyd, William R. Smith, Michael B. Edwards, Courtney L. Schultz, Perver Baran, Robin A. Moore, Nilda Cosco, North Carolina State University, Raleigh, North Carolina; Luis J. Suau, Shaw University, Raleigh, North Carolina.

## Abstract

**Introduction:**

Parks provide opportunities for physical activity for children. This study examined sex differences in correlates of park-based physical activity because differences may indicate that a standard environmental intervention to increase activity among children may not equally benefit boys and girls.

**Methods:**

The System for Observation Play and Recreation in Communities was used to measure physical activity among 2,712 children and adolescents in 20 neighborhood parks in Durham, North Carolina, in 2007. Sedentary activity, walking, vigorous park activity, and energy expenditure were the primary outcome variables. Hierarchical logit regression models of physical activity were estimated separately for boys and girls.

**Results:**

Type of activity area and presence of other active children were positively associated with boys’ and girls’ physical activity, and presence of a parent was negatively associated. A significant interaction involving number of recreation facilities in combination with formal activities was positively associated with girls’ activity. A significant interaction involving formal park activity and young boys (aged 0–5 y) was negatively associated with park-based physical activity.

**Conclusion:**

Activity area and social correlates of park-based physical activity were similar for boys and girls; findings for formal park programming, age, and number of facilities were mixed. Results show that girls’ physical activity was more strongly affected by social effects (eg, presence of other active children) whereas boys’ physical activity was more strongly influenced by the availability of park facilities. These results can inform park planning and design. Additional studies are necessary to clarify sex differences in correlates of park-based physical activity.

## Introduction

Most US children do not accumulate the recommended 60 minutes of daily physical activity, and adherence to recommendations is significantly lower among girls (1). Public parks and playgrounds are key components of environmental interventions to increase physical activity among children during nonschool hours and can be modified through public policy to further encourage daily physical activity (2).

Availability of a variety of recreation facilities and proximity to them have been associated with increased physical activity among adults (3) and children (4), with some exceptions (5). Improvements and renovations in parks (6) and school playgrounds (7) have been associated with increased park-based physical activity among children. Although studies have shown there are sex differences among youth park-based physical activity (8), research examining such differences in the context of social and environmental factors such as parental supervision and other children is limited (9).

Previous studies provide evidence that associations between environmental variables and physical activity vary between boys and girls (10,11). Sex differences present challenges for understanding how the built environment affords opportunities for physical activity among children. Such differences may indicate that a standard environmental intervention to increase activity among children may not equally benefit boys and girls. Understanding whether social and environmental determinants influence physical activity differently among boys and girls can be used to guide decisions related to programming options and the design and retrofitting of parks. Therefore, this study sought to determine if associations among social and environmental characteristics of parks and park-based physical activity among children varied by sex. We hypothesized that boys’ park use would result in greater intensity levels of park-based physical activity than girls’ park use across different park activity settings. Second, we hypothesized that social and environmental correlates of park-based physical activity vary between boys and girls.

## Methods

### Study settings

Direct observations were conducted in 20 neighborhood parks in Durham, North Carolina, in 2007. To ensure socioeconomic diversity of population and inclusion of neighborhoods with a mixture of races/ethnicities, this study focused on the mostly residential central area of the city. Simple random sampling was used to select 20 of 38 available parks. Mean park size was 10.3 acres (range, 0.5–45.9 acres). For each park, a 400-meter network buffer was constructed using ArcGIS 9.1 (Esri) to define park service areas (12,13). Park service areas are geographic catchment areas encompassing a population of potential users (12). Their mean size was 121.8 acres (range, 35.4–305.5 acres). Park service areas were used to identify contextual characteristics of study parks. Total population of census blocks within park service areas ranged from 32 to 1,796, with a mean of 780. African American residents were the largest racial/ethnic group (mean,55.1%; range, 1.4%–100%), followed by non-Hispanic white (mean, 36.1%, range 0%–96.9%) and Hispanic residents (mean = 9.7%, range 0%–32.4%). The mean percentage of children aged 18 years or younger was 24.3% and ranged from 16.0% to 35.7% (www.census.gov//census2000/states/nc.html).

### Measures

#### Physical activity

Park-based physical activity was measured using the System for Observing Play and Recreation in Communities (SOPARC) (14). Developed for open environments such as parks and playgrounds, SOPARC consists of systematic momentary time sampling of predetermined activity areas in parks. Park activity areas were scanned by trained observers visually sweeping from left to right, and the codes representing children’s activity levels were recorded on a standardized form along with other contextual information. Observations occurred for 8 weeks from 10AM to 7PM EDT on all weekend days and randomly selected weekdays in May, June, and July 2007. Each park was observed 16 times during the study period (8 weekend days and 8 weekdays). Observed activity was coded as sedentary (eg, standing, sitting, lying down), walking (eg, walking, other moderate intensity activities), or vigorous (eg, running, climbing, jumping) as validated in previous studies (15,16). The codes also provided estimates of energy expenditure rate (EER). EER was estimated by equating categories of activities with constants (sedentary, 0.051 kcal/kg/min; walking, 0.096 kcal/kg/min; very active, 0.144 kcal/kg/min) (17,18).

#### Demographic and social correlates

The SOPARC age category was modified to account for 3 age categories: young child (0–5 y), middle-child (6–12 y), and older children or adolescents (13–18 y). The observation codes for different age groups were introduced after the research staff was able to recognize and code SOPARC physical activity levels. Age was determined by observable physical and biological features (eg, height) and social context (eg, with a parent). The description of the training protocols are reported elsewhere (19). Paired observations from 4 study weeks produced data to assess interrater reliability for all physical activity codes for each age group. Adequate reliability was observed for physical activity codes using the 3 age groups (Table 1).

**Table 1:** Cohen’s κ Coefficients and Percentage Observer Agreement by Age Group, Sex, and Physical Activity Code, Durham, North Carolina, 2007

Child Sex, Age^a^, and Activity Level	κ Coefficient (% Observer Agreement)					
	Training (127 Paired Observations)	Week 1 (130 Paired Observations)	Week 4 (152 Paired Observations)	Week 5 (117 Paired Observations)	Week 7 (120 Paired Observations)	Week 8 (75 Paired Observations)
Female, YC, S	0.67 (93.7)	0.57 (96.2)	0.66 (98.7)	0.56 (94.0)	0.80 (97.5)	0.81 (97.3)
Female, YC, W	0.26 (92.1)	0.38 (95.4)	0.74 (98.7)	0.15 (92.3)	0.59 (98.3)	0 (98.7)
Female, YC, V	0.56 (93.7)	0 (99.2)	1.00 (100)	−0.01 (98.3)	0.66 (99.2)	0 (98.7)
Male, YC, S	0.63 (96.1)	0.39 (97.7)	0.80 (98.7)	0.84 (98.3)	0.79 (98.3)	0.49 (97.3)
Male, YC, W	0.77 (96.1)	0.71 (98.5)	0.80 (98.7)	0.59 (98.3)	0.56 (97.5)	0.66 (98.7)
Male, YC, V	0.65 (96.9)	1.00 (100)	0.80 (99.3)	0.24 (97.4)	0.80 (99.2)	1.00 (100)
Female, MC, S	0.68 (92.9)	0 (99.2)	0.66 (97.4)	0.81 (98.3)	0.95 (99.2)	0.87 (98.7)
Female, MC, W	0.44 (89.0)	0.49 (97.7)	0.63 (97.4)	0.66 (98.3)	1.00 (100)	0 (98.7)
Female, MC, V	0.49 (91.3)	0.66 (99.2)	0.63 (97.4)	0 (98.3)	1.00 (100)	(100)^b^
Male, MC, S	0.55 (89.0)	1.00 (100)	0.65 (97.4)	0.59 (96.6)	0.75 (96.7)	0.90 (98.7)
Male, MC, W	0.60 (91.3)	(100)^b^	0.66 (96.7)	0.66 (99.2)	0.87 (98.3)	0.66 (98.7)
Male, MC, V	0.51 (94.5)	0 (99.2)	0.93 (99.3)	0.49 (98.3)	0.56 (97.5)	(100)^b^
Female, OC, S	0.55 (96.9)	(100)^b^	0.66 (99.3)	0.66 (99.2)	0.66 (99.2)	1.00 (100)^b^
Female, OC, W	0.43 (96.1)	(100)^b^	0.89 (99.3)	(100)^b^	1.00 (100)^b^	(100)^b^
Female, OC, V	(100)^b^	(100)^b^	1.00 (100)	(100)^b^	0 (99.2)	(100)^b^
Male, OC, S	0.68 (94.5)	(100)^b^	0.50 (99.3)	0.83 (99.2)	0.66 (99.2)	0 (98.7)
Male, OC, W	0.66 (93.7)	(100)^b^	(100)^b^	0.62 (97.4)	0.66 (99.2)	−0.03 (93.3)
Male, OC, V	0.83 (97.7)	(100)^b^	0.66 (99.3)	0.80 (99.2)	(100)^b^	0 (98.7)

Abbreviations: MC, middle child; OC, older child; S, sedentary physical activity; V, vigorous physical activity; W, walking or moderately active physical activity; YC, young child.

a Young child = age 0 to 5 years, middle child = age 6 to 12 years, old child = age 13 to 18 years.

b No variability observed; therefore, a weighted κ cannot provide a meaningful test statistic.

Presence of an adult was coded by observers as 1) not present, 2) supervising adult (eg, teacher, coach), 3) parent or caregiver, or 4) don’t know (mean κ, 0.6). Presence of other active children was a dichotomous variable indicating the presence of other moderately or very active children in an activity area (1 = yes). Formality of play was measured using 4 attributes: no play observed, free play, informal organized play (eg, group playground play, pick-up soccer), and formal organized play (eg, individual or group athletic event) (mean κ, 0.8).

#### Park environment correlates

SOPARC observations were conducted in predetermined zones (park activity areas) in each park. The areas for SOPARC observations were mapped by 3 members of the research team. Mean acres for SOPARC zones was 0.35 (standard deviation, 0.45). Environmental features in each activity area were measured using the Environmental Assessment of Public Recreation Spaces (EAPRS) instrument (20). Audits for the presence of facilities and amenities were conducted for each SOPARC zone (N = 134) during daylight hours by 2 pairs of 2 raters working independently. Features serving as primary supports for physical activity were categorized as recreation facilities (eg, trails, playground equipment). Secondary features (eg, tables, benches) were treated as park amenities (21). The mean κ across audited features was 0.9. Counts of facilities and amenities in park activity areas were derived for subsequent analyses. Activity area types were categorized on the basis of their designated use. These included playgrounds, courts, fields, open areas, swimming pools, and picnic areas and shelters. Size of activity areas was measured by calculating the area of the polygons comprising the activity areas. All study procedures were approved by the North Carolina State University’s institutional review board.

#### Analysis

First, differences in energy expenditure among boys and girls were compared for different activity settings (eg, playgrounds, sport fields, courts). Second, sex-stratified logit models of park-based physical activity were estimated to examine whether different patterns of associations existed for boys and girls.

Analysis of variance was used to test for sex differences in intensity of physical activity associated with different park activity areas. Hierarchical generalized regression models stratified by sex were used to examine whether social and environmental correlates of park-based physical activity varied between boys and girls. Hierarchical generalized linear models (22) were estimated because of the hierarchical structure of the data (individual children within park zones) and the use of a 3-level ordinal dependent variable (sedentary, walking, and very active). To address the hypothesis related to the environmental characteristics of park activity areas, unconditional models (intercept only) were estimated to establish whether variation in individual park-based physical activity levels were significantly associated with differences in park activity areas. Independent variables of interest were examined by using fixed effects and odds ratios controlling for other model predictors. Sedentary served as the reference category in regression models. Within-level and cross-level interactions were also examined. Analyses were performed in May 2011 using SAS version 9.2 (SAS Corp).

## Results

Characteristics of park users and activity areas are shown in Table 2. During the study period, 2,712 children were observed, and 43.5% were girls (Table 2). Among girls, 50.4% were categorized in the 0 to 5 years age group, followed by 39.2% in the 6 to 12 years age group and 10.3% in the 13 to 18 years age group. Among boys, the 6 to 12 years age group was most frequently observed (42.3%) followed by 36.6% for the 0 to 5 years age group and 21.1% for the 13 to 18 years age group. Boys were more likely to be observed in informally and formally organized park activities (16.1 and 9.4, respectively, for boys vs 5.8 and 4.1, respectively, for girls). Girls were also more likely than boys to be observed in free play (68.1 vs. 53.5) (*χ* ^2^ = 108.8, *P* < .001).

**Table 2:** Characteristics of Children and Adolescents Using Parks and Park Activity Areas, Durham, North Carolina, 2007

Characteristic	Girls	Boys
**Park Users, n (%)**		
**Sex**		
Girls	1,180 (43.5)	—
Boys	—	1,532 (56.5)
**Physical activity level**		
Sedentary	662 (56.1)	765 (49.9)
Walking	370 (31.4)	558 (36.4)
Vigorous	148 (12.5)	209 (13.6)
**Age group**		
0–5 y	595 (50.4)	560 (36.6)
6–12 y	463(39.2)	648 (42.3)
13–18 y	122 (10.3)	324 (21.1)
**Style of play**		
No play	255 (21.9)	318 (21.0)
Free play	793 (68.1)	811 (53.5)
Informal organized	68 (5.8)	244 (16.1)
Formal organized	48 (4.1)	142 (9.4)
Parent/guardian present	660 (55.9)	684 (44.6)
Supervising adult present	235 (19.9)	384 (25.1)
**Park areas**		
**Sex of park area users**		
Girls only or boys and girls, no. of park areas	89	—
Boys only or boys and girls, no of park areas	—	95
**Zone type**		
Playground	24 (26.9)	24 (25.3)
Courts	14 (15.7)	15 (15.8)
Fields	7 (7.8)	10 (10.5)
Trail/walking path	11 (12.4)	12 (12.6)
Shelter/picnic area	14 (15.7)	14 (14.7)
Open space	11 (12.4)	15 (15.8)
Other	8 (8.9)	5 (5.3)
**Areas with other active children present, %**	23.8	26.3
**Zone size, sq ft, mean (SD)**	15,966 (20,499)	15,938 (19,279)
**No. of facilities, mean (SD)**	1.1 (0.8)	1.1 (0.8)
**No. of amenities, mean (SD)**	1.3 (1.5)	1.2 (1.4)

Abbreviation: SD, standard deviation.

Regarding supervision, a parent or guardian was more likely to be present among girls (55.9%, girls vs 44.6%, boys). Presence of other adult supervisors was more likely to be observed among boys (25.1%, boys vs 19.9%, girls). Activity levels were similar across boys and girls, with boys slightly more active than girls overall. Of girls, 56.1% were observed in sedentary activity compared with 49.9% of boys. Of girls, 31.4% were observed in walking activity behaviors compared with 36.6% of boys.

### Results of hierarchical generalized regression model 

#### Girls’ park-based physical activity

Examination of the fixed effects indicated that significant variation existed in thresholds (intercepts) across all park activity areas between sedentary activity and vigorous activity (intercept 1) but not between sedentary and walking (intercept 2) (Table 3). Controlling for predictor variables, girls across all park activity areas were equally likely to be observed sedentary as they were to be observed walking (moderate intensity activities).

**Table 3:** Hierarchical Cumulative Logit Model for Park-Based Physical Activity of Children and Adolescents, Durham, North Carolina, 2007

Characteristic	Girls (Level 1 N = 1,180, Level 2 N = 87)			Boys (Level 1 N = 1,532, Level 2 N = 95)		
	Estimate (SE)	*P* Value	Odds Ratio (95% CI)	Estimate (SE)	*P* Value	Odds Ratio (95% CI)
**Fixed Effects**						
**Main effects**						
Intercept 1^a^(vigorous activity)	−1.91 (0.461)	<.001	—	−2.32 (0.374)	<.001	—
Intercept 2^a^(walking activity)	0.172 (0.456)	.70	—	−0.124 (0.368)	.73	—
Age category 1 (0–5 y)	0.438 (0.394)	.26	1.54 (0.77–2.31)	0.338 (0.307)	.27	1.4 (0.79 to 2.00)
Age category 2 (6–12 y)	0.336 (0.395)	.39	1.39 (0.62– 2.16)	0.151 (0.282)	.59	1.16 (0.60 to 1.71)
Age category 3^b^ (13–18 y)	1[Reference]					
Parent present	−0.756 (0.193)	<.001	0.469 (0.09–0.85)	−0.423 (0.172)	.01	0.655 (0.32 to 0.99)
Supervising nonparental adult present	−0.322 (0.22)	.14	0.725 (0.29–1.16)	−0.339 (0.178)	.05	0.712 (0.36 to 1.06)
Formality of play	−0.251 (0.314)	.42	0.778 (0.16–1.39)	0.203 (0.202)	.31	1.23 (0.83 to 1.63)
**Level 1 interaction, by age^b^**						
Age 1 × formality of play	−0.164 (0.334)	.62	0.849 (0.19–1.50)	−0.46 (0.215)	.03	0.631 (0.20 to 1.06)
Age 2 × formality of play	−0.229 (0.336)	.49	0.795 (0.14–1.45)	−0.17 (0.194)	.38	0.806 (0.46 to 1.22)
Age 3 × formality of play^b^	1[Reference]					
**Park activity area characteristics**						
Zone size, area in ft^2^	3.19E–06 (7.29E–06)	.66	1 (0.99–1.00)	−6.76E–06 (−6.12E–06)	.27	1 (−0.99 to 1.00)
Recreational facilities	−0.394 (0.211)	.06	0.674 (0.26–1.09)	0.099 (0.2)	.62	1.1 (0.71 to 1.49)
Park amenities	−0.025 (0.107)	.81	0.975 (0.76–1.18)	0.022 (0.098)	.81	1.02 (0.83 to 1.21)
Other active children in area (1= yes)	1.58 (0.167)	<.001	4.85 (4.52–5.18)	1.14 (0.143)	<.001	3.12 (2.83 to 3.40)
Zone type						
Picnic/shelter	−1.03 (0.545)	.05	0.357 (−0.71 to 1.43)	−1.22 (0.334)	.01	0.295 (−0.36 to 0.94)
Courts	0.253 (0.436)	.56	1.29 (0.44–2.14)	0.746 (0.337)	.02	2.11 (1.45 to 2.77)
**Cross-level interaction**						
Recreational facilities × formality of play	0.343 (0.109)	.002	2.21 (1.99–2.42)	0.032 (0.107)	.77	1.03 (0.82 to 1.24)
**Variance components**						
Level 2 variance (τ_00_)	0.793 (0.255)	—	—	0.648 (0.192)	—	—
**Goodness of fit (independence)**						
−2 log L	9,205.41	—	—	11,757	—	—
Independence −2 log L	9,251.73	—	—	11,805.15	—	—
χ^2^	46.32	<.001	—	48.15	<.001	—

Abbreviations: E, exponent value; SE, standard error; —, not applicable

a Sedentary was used as reference category for the ordinal outcome variable.

b Age 1 = 0 to 5 years; age 2 = 6 to 12 years; age 3 = age 13 to 18 years. Age category 3 was used as reference category.

**Individual level effects (level 1) and cross-level interactions.** Within park activity areas and controlling for other variables in the model, the presence of a parent was associated with lower odds (odds ratio [OR], 0.47) of an increased level of physical activity for girls. The presence of other active children in a park activity area had the strongest positive association (OR, 4.85) with girls’ park-based physical activity. A significant positive interaction involving the number of recreation facilities and formally organized activity was associated with 2.21 increased odds of higher park-based physical activity. That is, girls participating in more formal and organized play had higher odds of increased physical activity in activity areas with a greater number of recreation facilities.

#### Boys’ park-based activity

Examination of the fixed effects indicated that significant variation existed in thresholds (intercepts) across park activity areas between sedentary and vigorous activity (intercept 1) but not between sedentary and walking (intercept 2). Controlling for predictor variables, boys across park activity areas were no less likely to be observed sedentary as they were to be observed walking.

**Individual level effects (level 1) and cross-level interactions.** As with girls, but to a lesser extent, the presence of a parent was associated with a lower likelihood (OR, 0.66) of high levels of physical activity. A stronger interaction of age and formality of play associated with lower odds of increased physical activity was found among boys in this sample in comparison with girls. Among boys categorized as age 0 to 5, each increased level of formalization and organization in park activity was associated with a 37% decrease in odds of higher levels of physical activity. Boys observed in park activity areas identified as picnic areas and shelters were 70% less likely to be engaged in high levels of physical activity than boys in other types of park activity areas. High levels of park-based physical activity were associated with courts (eg, basketball, tennis, etc.) and presence of other active children.

In sum, high levels of girl’s park-based activity were associated with presence of other active children and a combination of formal activity and an increasing number of recreation facilities in park activity areas. Common correlates between girls and boys were presence of a parent and presence of other active children and a low likelihood of increased activity associated with picnic areas. Among boys, increased activity was also associated with athletic courts.

## Discussion

Neighborhood parks have potential to help children accumulate recommended levels of daily physical activity. This study sought to determine whether social and environmental correlates of park-based physical activity differed between boys and girls. Multivariate sex-specific models provided evidence of similarities and noteworthy differences in social and environmental correlates for boys and girls. Models for boys and girls showed that the type of park activity area was associated with increased likelihood of vigorous activity but not with walking intensity activity. This finding is important in 3 respects. First, it indicates that characteristics of park activity areas may be uniquely associated with vigorous activity, a “relative rare” intensity level (1). Second, previous studies indicate that vigorous intensity is more strongly associated with youth fitness levels and weight status (23,24). Finally, such findings provide direction for identifying appropriate spatial scales for park-based physical activity interventions. Interventions can target multiple scales (ie, neighborhoods, parks, and areas within parks). In light of data from earlier studies (6–9), these findings suggest that activity areas within parks can be the focus of environmental or policy change.

Relationships between social correlates and physical activity were similar for boys and girls; however, noteworthy differences emerged. Presence of other active children had a stronger positive association with park-based physical activity than any other predictor variable. The association was stronger among girls where presence of other active children increased the odds of higher activity levels by 4.85 times compared with 3.12 times for boys. Methods used in the current study precluded assessment of relationships among children. The findings are nevertheless consistent with studies that show friendships among adolescents are strong predictors of their physical activity levels (9). Presence of parents was associated with a lower likelihood of increased activity among children and adolescents with a stronger negative trend observed among girls. These findings support the hypothesis that gender norms affect how some parents respond to children’s behavior. Possibly parents’ efforts to supervise and monitor their children’s safety inadvertently curtails higher levels of activity. The pervasiveness of gender norms may encourage some parents to be more protective of girls and encourage “safer” sedentary play activities. Other research suggests parental anxieties about safety are more constraining of young children’s physical activity than the level of public recreation facilities (25). Communicating with parents about the social and physiologic importance of active play is a major challenge in children’s health promotion (26,27). The results suggest that active play among girls should be emphasized.

Differences between boys and girls were observed for interactions involving formality of park activities. Among boys in the 0 to 5 age group, formal park activity was associated with a low level of physical activity. Thus, park programming designed to promote physical activity for young boys could minimize formal games and emphasize free play (26). By contrast, among girls, formal park activity and a greater number of recreation facilities appeared to increase high levels of physical activity. Timperio et al (28) reported that an increased number of neighborhood recreation facilities was inversely associated with walking activity among young girls. Our findings suggest that programming and availability of facilities within parks appear to be more important for girls than boys among all age groups. This may be because parents are more reluctant to let girls play outside if unsupervised (29) and may be less reluctant if programming and facilities are available because of their association with supervision.

Strengths of this study include use of sex-stratified models to examine social and environmental correlates of park-based physical activity; measurement of 3 different age groups of children; use of validated assessment tools (SOPARC and EAPRS) to measure actual park use, park-based physical activity, and environmental features; statistical analyses adjusted for clustering within park activity areas; and parks sampled from racially diverse neighborhoods. Primary limitations were that observations of children occurred at 1 point in time and not continuously during the course of children’s visit to the park; energy expenditure was not measured directly; the study design was cross-sectional; we did not know whether parks users were demographically similar to the park service area population; and results were not generalizable beyond the summer season.

To our knowledge this is the first study using sex-stratified models to examine social and environmental correlates of park-based physical activity among children and adolescents. Overall, similar patterns of associations were observed for boys and girls. Characteristics of activity areas, presence of parents, and presence of other active children exhibited similar effects. Key differences were type of activity areas among boys, formality of park activity among young boys, and presence of recreation facilities interacting with formality of park activity among girls. Public parks and recreation facilities are important community resources for promoting physical activity (2). To maximize parks’ contribution to children’s total physical activity, future studies should build on these results to identify and evaluate social and environmental factors with strong potential to support walking and vigorous activity levels within parks. Parks are built environmental features with high relevance for children (13,30), and more studies are needed to learn how the children’s sex influences the effect of social and built environment correlates on park-based activity.

Park-program specialists and researchers should consider the role parents and other children play in encouraging activity. Our results indicate that parents may discourage high intensity activity among children with a more noticeable influence among girls. Presence of other active children appears conducive to higher intensity activity, especially for girls. Presence of specific park facilities should also be considered. Courts were more conducive for boys’ activity. However, picnic areas were less conducive to physical activity among both boys and girls. A combination of organized park activities and recreation facilities produced greater activity among girls.

These findings suggest that environmental correlates that support physical activity in parks differ across age groups and between girls and boys. Enhanced knowledge about social and environmental factors that increase the frequency and intensity of physical activity among children should help park administrators, programmers, and planners to design parks that meet the needs of children of all ages and of both sexes. To better understand how parks benefit children’s physical activity, potential differences in the characteristics of park environments that encourage or discourage boys’ and girls’ physical activity should be further examined.
